# The effect of locomotive syndrome on the trajectory of sleep disturbance in geriatric oncology inpatients

**DOI:** 10.3389/fonc.2025.1440747

**Published:** 2025-02-03

**Authors:** Yu-Ling Yang, Hui Su, Hui Lu, Jing Wang, Yu-Qing Zhou, Ling Li, Hui Yu, Yan Lv, Ying Chen

**Affiliations:** ^1^ Department of Oncology, Affiliated Hospital of Jiangnan University, Wuxi, Jiangsu, China; ^2^ Medical Check-up Center, Taihu Sanatorium of Jiangsu Province, Wuxi, Liaoning, China

**Keywords:** geriatric oncology inpatients, sleep disturbance trajectory, locomotive syndrome, cancer epidemiology and prevention, circadian rhythm and health

## Abstract

**Objective:**

To explore the developmental trajectory of sleep disturbance in geriatric oncology inpatients and assess the impact of locomotive syndrome (LS) on this trajectory.

**Methods:**

This longitudinal study enrolled 284 geriatric oncology inpatients through convenience sampling from August 2023 to February 2024 at the Oncology Center of the Affiliated Hospital of Jiangnan University, Wuxi, Jiangsu Province, China. Sleep quality was monitored for seven days following admission (T0-T6) using a wrist motion analyzer. The developmental trajectory of sleep disturbance was analyzed using the latent category growth model (LCGM). Patients were categorized into the LS group (n=177) and the non-LS group (n=107) based on the 25-question Geriatric Locomotive Function Scale (GLFS-25). Comparisons were made between the two groups regarding the distribution of sleep disturbance trajectories.

**Results:**

Three potential categories for the development trajectory of sleep disturbance in inpatients were identified: non-sleep disturbance, sleep disturbance improvement, and sleep disturbance persistence. The lowest sleep quality was observed on the second day after admission (T2). In the non-LS group, 40 cases (37.4%) experienced no sleep disturbance, 45 cases (42.1%) showed improvement, and 22 cases (20.6%) showed persistence of sleep disturbance. In the LS group, 32 cases (18.1%) experienced no sleep disturbance, 50 cases (28.2%) showed improvement, and 95 cases (53.7%) exhibited persistent sleep disturbance. Significant differences were found in the trajectory distribution of sleep disturbance between the two groups (*P*<0.001).

**Conclusions:**

Sleep disturbance is prevalent in geriatric oncology inpatients, with an incidence of 74.6% (212/284), and is most severe on the third day after admission. Patients with LS exhibit lower overall sleep quality and a higher likelihood of persistent sleep disturbances.

## 1 Introduction

Surveys conducted by the National Institutes of Research in the United States indicate that age is the primary risk factor for tumorigenesis (1). Recently, the incidence of malignant tumors in the elderly has increased annually. The National Oncology Center’s 2020 report revealed that there were 19.29 million new cases of tumors worldwide and 4.57 million in China, predominantly among individuals aged 60 to 79 years (2).

With the gradual decline of physiological functions in the elderly, changes occur in sleep duration, circadian rhythms, and sleep efficiency. These changes are characterized by prolonged latency to fall asleep, reduced duration of deep sleep, sleep fragmentation, and tendencies toward early bedtime and waking (3). Studies indicate that the prevalence of sleep disturbances in the elderly ranges from 30% to 40% (3), with figures in China’s elderly population varying between 29.5% and 63.3% (4). Sleep disturbances impair the body’s ability to rest and recover, leading to insufficient blood supply and reduced immunity, metabolism, and other functions (5). Furthermore, geriatric oncology patients, who often undergo various treatments and take multiple medications, experience reduced sleep quality, which can exacerbate cognitive dysfunction, pain sensitization, cardiac arrhythmias, blood pressure fluctuations, and other complications, potentially leading to accidental deaths (6).

The Japanese Orthopaedic Association (JOA) has developed the concept of “Locomotive Syndrome (LS)” to help residents recognize the deterioration of musculoskeletal function (MF), which increases with age (7, 8). LS is characterized as a high-risk state for restricting normal life due to degenerative dysfunction of one or more parts of the musculoskeletal system (9, 10). The components of the musculoskeletal system involved in LS include: (1) bones; (2) joints and intervertebral discs; (3) muscles and nerves (10, 11). Progression of LS can result in: (1) osteoporosis and associated fractures; (2) osteoarthritis and spondylosis; (3) sarcopenia and neurological disorders (11). These conditions may lead to pain, limited joint mobility, poor posture, and imbalance, which in turn can cause difficulty in standing and walking, further deterioration of MF, and ultimately a dependency on caregiving (8, 9). LS susceptibility is markedly pronounced in geriatric tumor survivors. Factors such as advanced age, tumors, and the associated treatments and convalescence that necessitate prolonged bed rest contribute to muscle wasting, atrophy, and joint contractures. Additionally, interventions like surgery, radiotherapy, or chemotherapy can cause secondary complications like osteoporosis, peripheral neuropathy, lymphedema, an enhanced inflammatory response, and metabolic derangement of skeletal muscle. These factors significantly heighten the risk and severity of LS in elderly cancer patients.

In recent years, studies have increasingly examined the potential correlation between sleep disturbance and MF disorder in the elderly. American researcher Ensrud conducted a large-scale community survey on the sleep patterns of elderly individuals and discovered that those with movement disorders experienced more pronounced sleep disturbances than those without such disorders (12). A significant cohort study demonstrated that the prevalence and severity of MF disorders were considerably higher among elderly patients with sleep disturbances compared to those without, indicating that sleep disturbances may contribute to decreased muscle strength and balance, reduced mobility, increased fall risk, and cognitive decline in this population (13–15).

Previous research has shown that geriatric oncology patients, due to advanced age and intensive antitumor therapy, exhibit a high prevalence of reduced sleep quality and MF disorders, which may adversely affect their prognosis (16, 17). Current research on the link between sleep disturbance and MF disorder primarily involves cross-sectional studies. There is a scarcity of studies exploring the dynamic development of this relationship, highlighting the need for research into the dynamic interaction between sleep and MF disorders. This study aims to trace the progression of sleep quality changes in geriatric oncology inpatients post-admission and analyze the impact of LS on the developmental trajectory of sleep disturbances. This could offer new insights for interventions targeting sleep disturbances in these patients, potentially conserving social and public healthcare resources and lessening the caregiver burden, which holds significant social and economic value.

## 2 Materials and methods

The study received approval from the Ethics Committee of the Affiliated Hospital of Jiangnan University (*No*. LS2023101) and was registered with the Chinese Clinical Trial Registry (*No*. ChiCTR2400079958). All participants provided signed informed consent. This study adhered to the standards set forth in the Declaration of Helsinki.

### 2.1 Objects

Geriatric oncology patients hospitalized in the Department of Medical Oncology at the Affiliated Hospital of Jiangnan University from August 2023 to February 2024 were selected using a convenience sampling method.

**Inclusion criteria:** (1) Age≥60 years; (2) Diagnosed with malignant tumors according to the eighth edition of the International Tumor TNM staging criteria, without bone metastasis, as confirmed by a physician; (3) Previously good sleep quality, indicated by a Pittsburgh Sleep Quality Index (PSQI) score<7; (4) Ability to communicate effectively, possessing adequate reading and writing skills, and capable of completing questionnaires; (5) Voluntary participation and signed informed consent.

**Exclusion criteria:** (1) Hospitalization for less than 7 days; (2) Presence of acute episodes or emergencies related to respiratory, cardiovascular, or cerebrovascular conditions, such as asthma or acute myocardial infarction; (3) Unstable tumor condition undergoing intensive cancer treatment; (4) Serious physical or psychological illnesses, such as depression; (5) Mental and behavioral disorders.

### 2.2 Research tools

#### 2.2.1 General information questionnaire

This questionnaire collects data on inpatients’ gender, age, height, weight, marital status, tumor type, disease duration, metastatic recurrence, radiotherapy, and comorbidities.

#### 2.2.2 LS

The 25-question Geriatric Locomotive Function Scale (GLFS-25), developed by Japanese scholar Seichi, was used to assess LS. The GLFS-25 includes 25 items across four dimensions: physical pain, activities of daily living, social activities, and mental status. Each item is scored on a Likert’s 5-point scale (0-4 points). The scale has a Cronbach’s *α* coefficient of 0.93, indicating high reliability and validity. Patients scoring≥16 were categorized in the LS group, and those scoring <16 in the non-LS group.

#### 2.2.3 Pittsburgh sleep quality index

The PSQI consists of 19 self-assessed and 5 other-assessed items, covering seven components: sleep quality, time to fall asleep, sleep duration, sleep efficiency, sleep disturbances, use of hypnotic drugs, and daytime functioning. Each item is scored on a 4-point Likert scale (0-3), with the total PSQI score indicating the presence of sleep disturbance (a score of ≥7 suggests sleep disturbances, with higher scores indicating poorer sleep quality).

#### 2.2.4 Sleep disturbance trajectory monitoring methodology

Sleep was monitored using a wrist actigraph (model: wActi-Sleep BT monitor) manufactured by Actigraph GmbH, Germany. Upon enrollment, researchers provided individual education and guidance on how to monitor sleep quality, including the initial setup of the wrist actigraph (e.g., entering patient number, bedtime, wake-up time). Patients completed treatments and daily activities by 20:00 during hospitalization and were instructed to wear the wrist actigraph when going to sleep. The actigraph measured sleep latency, total sleep time, sleep efficiency, and other indicators based on wrist movement, using the Cole-Kripke algorithm to calculate sleep quality scores from 0 to 100, where higher scores indicate better sleep quality. Researchers accessed sleep quality scores via cellphone. Sleep monitoring commenced on the first day of hospitalization and continued for 7 consecutive days (T0 to T6).

### 2.3 Sample size

A sample size of 300 was determined through a literature review, referencing longitudinal trajectory-related studies with sample sizes ranging from 6 to 303 cases, and accounting for a projected dropout rate of 10% to 20%.

### 2.4 Methods of data collection

Researchers explained the purpose and significance of the study in detail to the inpatients at the project’s inception. After ensuring subjects’ understanding, an informed consent form was signed. All patients were enrolled and completed the questionnaires under the researcher’s guidance. Questionnaires were collected immediately upon completion, and any missing or omitted items were promptly addressed.

### 2.5 Statistical methods

Statistical analysis was conducted using SPSS 19.0 and Mplus 7.0 software. Count data were reported as the number of cases and percentage. The *χ^2^* test was used for group comparisons. Normally distributed measurement data were presented as mean ± standard deviation ( $\bar{X}$ ± s), and the *t-*test was applied for comparisons between two groups. Latent class growth models (LCGM) identified the sleep disturbance trajectory categories and characteristics of the patients. Fit indicators including the Akaike information criterion (AIC) and Bayesian information criterion (BIC), entropy value, category probability, Vuong-Lo-Mendell-Rubin likelihood ratio test (VLMR), and Bootstrapped Likelihood Ratio Test (BLRT) were used to compare model fits. A better model fit is indicated by smaller AIC and BIC values. When VLMR and BLRT values reached a significant level (<0.05), the model with k categories was considered significantly better than the model with k-1 categories; a higher entropy value indicated more accurate classification, deemed accurate if the value was >0.8. P < 0.05 was considered statistically significant.

## 3 Results

### 3.1 Basic information of 284 geriatric oncology inpatients

**Figure 1:**
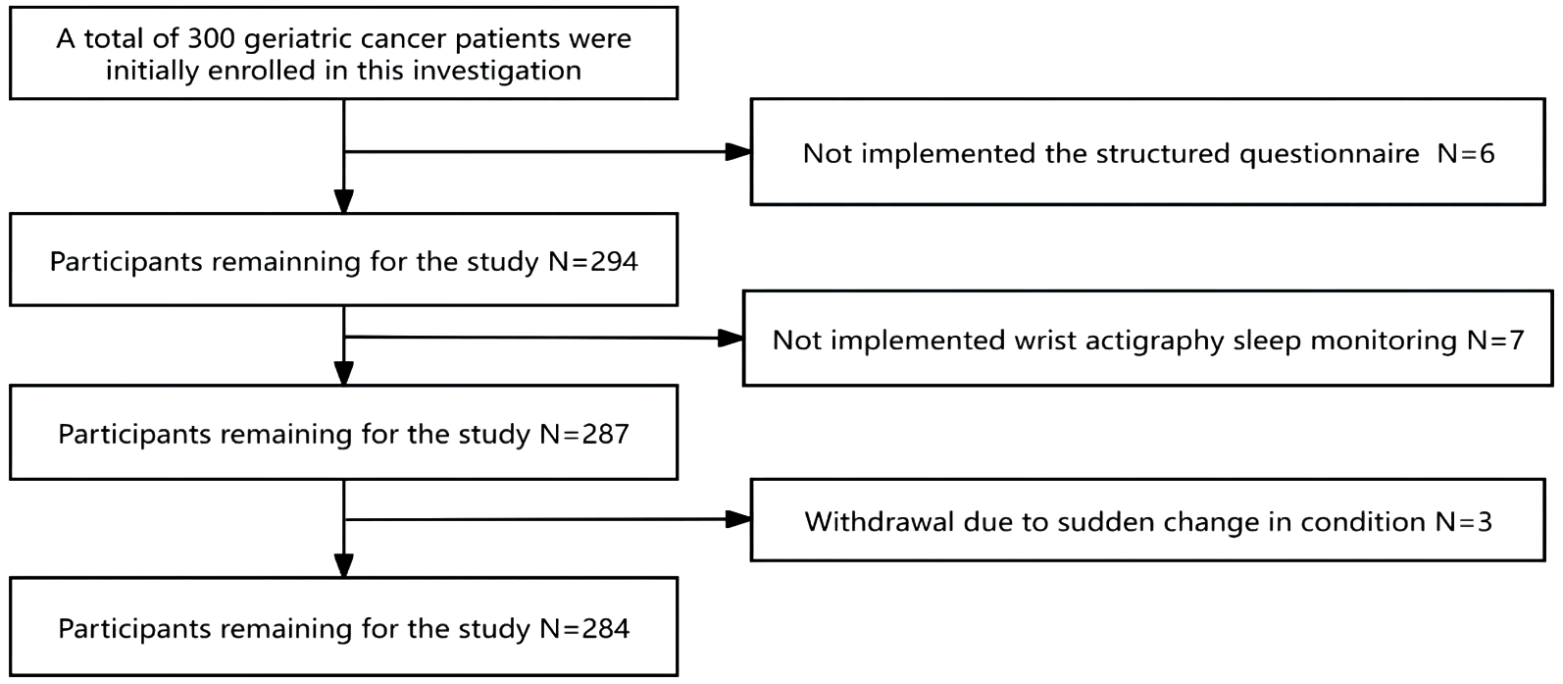
Flow chart of study participants.

**Figure 2:**
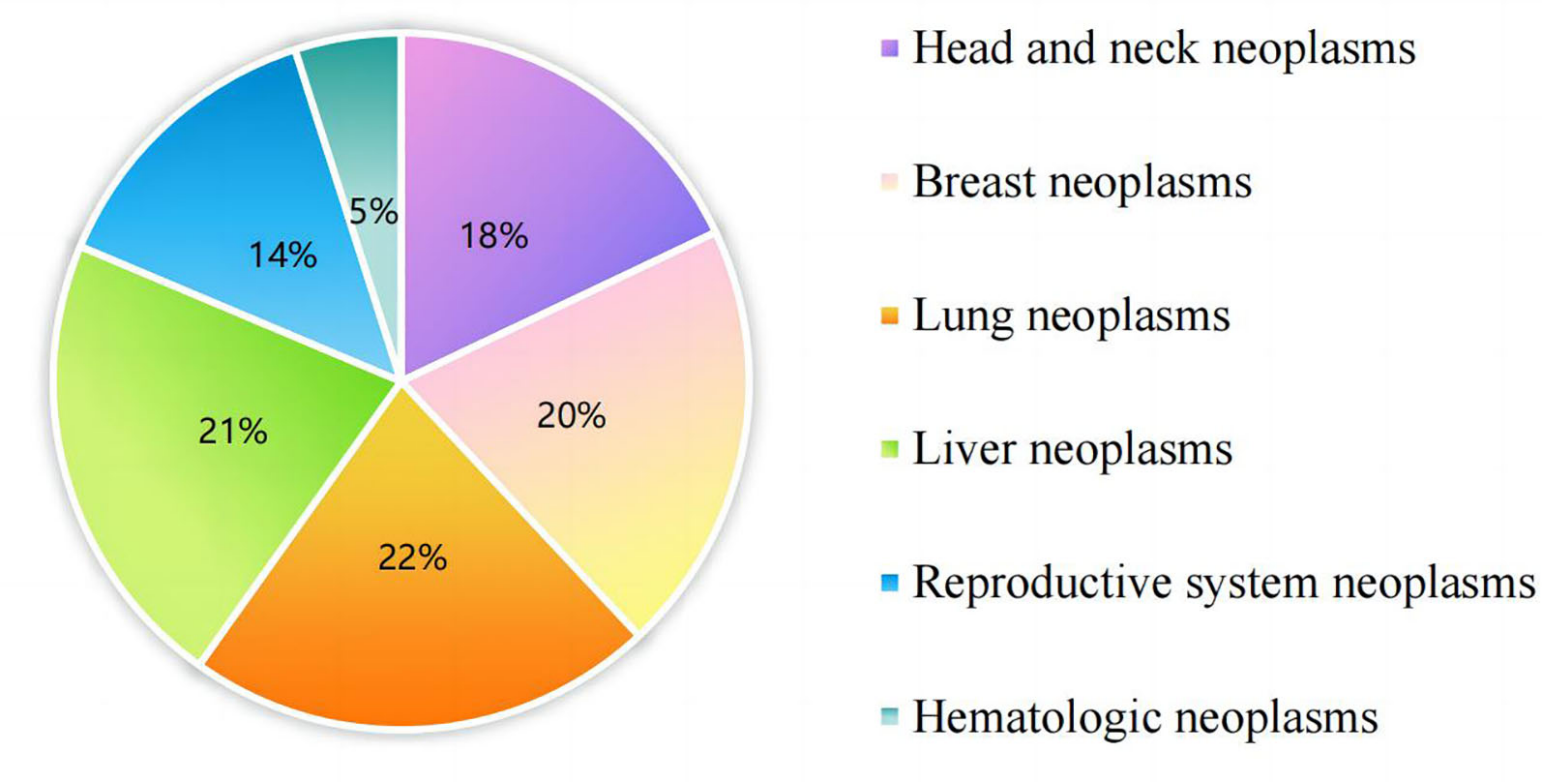
Types of primary tumor of 284 geriatric oncology inpatients.

Originally, 300 geriatric oncology inpatients were recruited for the study. Exclusions included 6 subjects who were unable to complete the questionnaire, 7 who failed to complete ambulatory sleep monitoring, and 3 who could not continue due to a sudden change in their condition, leaving data from 284 subjects for analysis. The demographic flowchart is depicted in **Figure 1**. The average age of the inpatients was 67.15 ± 6.33 years old, ranging from 60 to 74 years old. The mean duration of oncology disease was 5.25 ± 2.35 years. The mean GLFS-25 score was 15.73 ± 4.56, with 177 patients categorized in the LS group and 107 in the non-LS group. The distribution of primary tumor types is shown in **Figure 2**. Of the participants, 67 (23.7%) had no comorbid chronic diseases, 173 (61.0%) had 1 to 2 comorbidities, and 44 (15.3%) had 3 or more chronic diseases. Detailed demographic data are provided in **Table 1**.

**Table 1:** Comparison of the occurrence and severity of LS in oncology survivors with different characteristics.

Items	Groups	LS (N=177)	None LS (N=107)	t/x2	P
Age (Years)		67.05+5.98	67.26+7.01	-0.798	0.424
Gender	Male	90	50	0.517	0.605
	Female	87	57		
BMI		22.52+1.61	22.241.50	1.632	0.108
PSQI score		4.15+1.02	3.94+0.99	1.624	0.109
Radiotherapy	None	95	59	1.654	0.575
	Yes	82	48		
Chemotherapy	None	154	95	1.231	0.786
	Yes	23	12		
Recurrence	None	137	88	1.713	0.276
	Yes	30	19		
History of Falling	None	169	103	1.334	0.453
	Yes	8	4		
Visual Abnormality	None	153	97	0.893	0.312
	Yes	24	10		
Auditory	None	157	93	0.986	0.564
Abnormality	Yes	20	14		
Somatosensory	None	158	101	1.324	0.675
Abnormality	Yes	19	6		

PSQI, Pittsburgh Sleep Quality Index.

### 3.2 Developmental trajectory of sleep disturbance in 284 geriatric oncology inpatients

**Figure 3:**
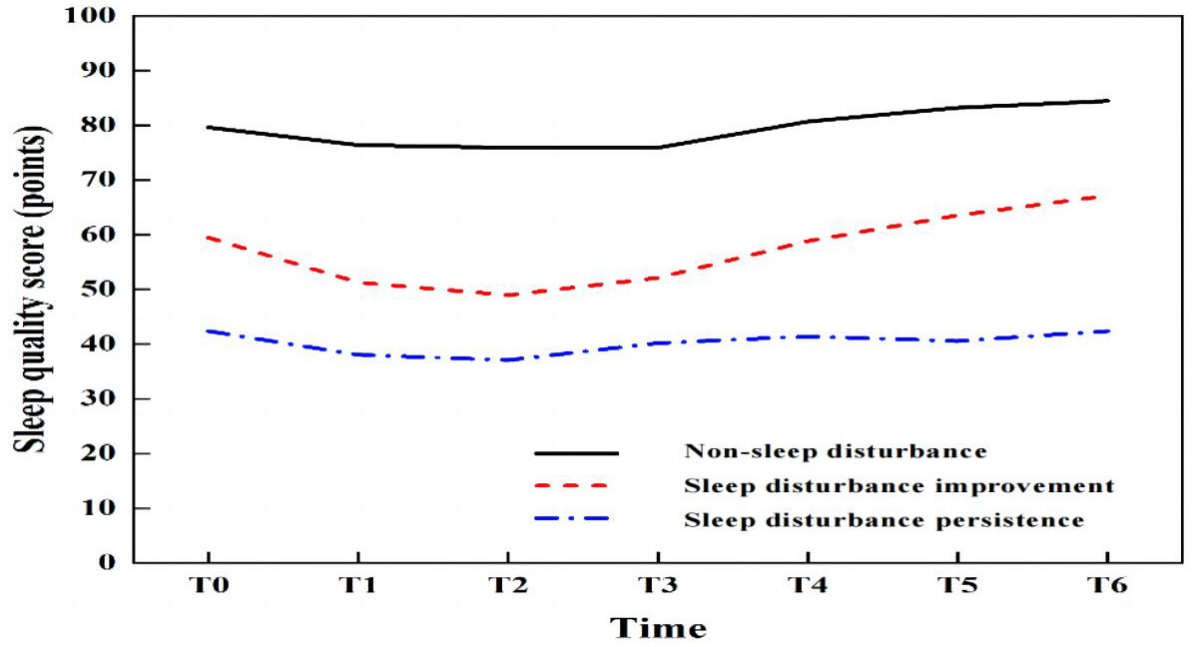
Trajectory of the latent class growth model (LCGM) for sleep disturbance in 284 geriatric oncology inpatients.

Trajectory analysis of sleep quality scores over seven time points from T0 to T6 was conducted using LCGM model fitting, with category parameters ranging from 1 to 6. Both AIC and BIC decreased with the increase in the number of categories, and significant levels were achieved in category 4 (*P* < 0.01), with an entropy value > 0.8, indicating that category 4 is the optimal model for LCGM detailed data are provided in **Table 2**. Using this model, patients were divided into three potential categories: non-sleep disturbance (72 cases, 25.4%), sleep disturbance improvement (95 cases, 33.5%), and sleep disturbance persistence (117 cases, 41.2%). The trajectory analysis revealed that the lowest sleep quality scores occurred at T2, with detailed information presented in **Figure 3**.

**Table 2:** Results of LCGM model fitting for sleep quality scores in 284 geriatric oncology inpatients.

Categories	AIC	BIC	Entropy	VLMR	BLRT	Categorical probability					
						1	2	3	4	5	6
1	6053.2	6098.2	-	-	-	0.332	-	-	-	-	-
2	5856.6	5794.7	0.911	0.052	<0.001	0.192	0.282	-	-	-	-
3	5657.2	5687.6	0.931	0.071	<0.001	0.292	0.172	0.396	-	-	-
4	5462.4	5598.6	0.942	<0.001	<0.001	0.223	0.275	0.458	0.365	-	-
5	5399.7	5389.8	0.929	0.263	<0.001	0.197	0.256	0.234	0.356	0.289	-
6	5137.5	5207.8	0.927	0.376	<0.001	0.329	0.293	0.257	0.361	0.271	0.18

LCGM, Latent class growth models; AIC, Akaike information criterion.

BIC, Bayesian information criterion; VLMR, Vuong-lo-mendell-rubin likelihood ratio test.

BLRT, Bootstrapped likelihood ratio test; "-", Duplicate data.

### 3.3 Differences in the distribution of sleep disturbance trajectories in geriatric oncology inpatients with different LS statuses

**Figure 4:**
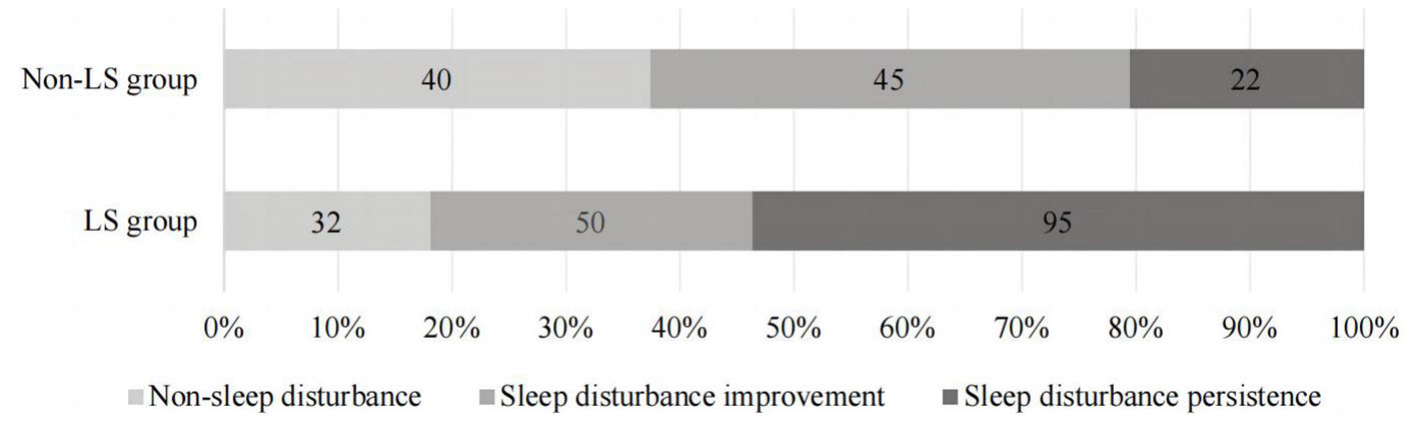
The distribution of sleep quality in geriatric oncology inpatients with different LS status.

The distribution of sleep quality among geriatric oncology inpatients with varying LS statuses is illustrated in **Figure 4**. The differences in the distribution of sleep disturbance trajectories between the two groups were statistically significant (*P* < 0.01). Detailed data are provided in **Table 3**.

**Table 3:** Distribution of sleep disturbance trajectories in geriatric oncology inpatients with different LS statuses.

Groups	N	Non-sleep disturbance	Sleep disturbance Sleep disturbance improvement	Sleep disturbance persistence
Non-LS Group	107	40	45	22
LS Group	177	32	50	95
χ^2^	98.465			
P	<0.001			

## 4 Discussion

The body’s MF gradually declines with age. MF encompasses more than just motor incapacity due to muscular, skeletal, or joint diseases, it also includes a decline in the ability to perform daily activities, and a decrease in somatic function and interest in activities (18). LS represents a broader concept of MF decline. It is focusing more on evaluating a subject’s overall MF rather than specific symptoms such as sarcopenia or weakness (19, 20). Degradation of motor organs, such as musculoskeletal organs, usually occurs very slowly and is difficult to detect, LS may be present even when the individual is not experiencing obvious debilitation or sarcopenia (19, 21). Consequently, this study employed LS to assess MF in 284 geriatric oncology inpatients.

### 4.1 Trajectory of sleep disturbance in geriatric oncology inpatients

Sleep plays a crucial role in metabolic homeostasis, memory retention, and brain function in biological organisms, and is intricately linked to an individual’s physiological, mental health, and social functioning. This study indicates that geriatric oncology inpatients are more susceptible to sleep disturbances at the early stage of hospital admission. Numerous factors contribute to sleep disturbances, with some scholars advocating for the “3P” hypothesis, which categorizes these factors into susceptibility (personality, motor status, age, etc.), predisposing (stressful life events or work, somatic conditions, medications, etc.), and sustaining factors (poor sleep habits, occupation, etc.). Sleep disturbances can emerge from any single category, but persistent insomnia typically results from an interplay of all three factors (22). Sleep disturbance are commonly reported during hospitalization, with prevalence rates between 47% and 67% in international studies (23, 24). In China, the incidence among geriatric inpatients ranges from 50% to 60% (25). In this study, the LCGM was employed to analyze the developmental trajectory of sleep quality in 284 hospitalized patients. The results indicated that 25.4% (72/284) of patients experienced no sleep disturbance, while the remaining 74.6% (212/284) exhibited improved or persistent sleep disturbances. The prevalence of sleep disturbances was notably higher among geriatric oncology inpatients, likely due to a combination of chronic physical and psychological conditions and the impact of the disease and medication on sleep, thereby increasing the risk of sleep disturbances in this demographic. It is important to note that the monitoring and observation of sleep quality in this study were concentrated during the first week of hospitalization. This focus was chosen because sleep disturbances are most pronounced in this initial period and to prevent the incomplete collection of data due to patient discharge.

This study employed wrist actigraphy to monitor sleep quality, offering a more objective and accurate assessment than the PSQI. Most sleep quality scales typically reflect sleep over the past month and fail to capture immediate changes in sleep status and trajectories during hospitalization. Analysis of the development of sleep disturbances from admission day (T0) to the sixth day (T6) revealed the lowest sleep quality scores on the third day (T2), aligning with the findings of Lee, which noted the peak of patient discomfort from the time of hospitalization to the third day (26). Hospitalization, considered a significant life stressor, often triggers psychological and motor changes in patients. Additionally, the hospital environment—characterized by shared rooms, noise from conversations, snoring, and medical equipment—can contribute to patient insecurity. Studies have indicated that the impacts of physical, psychological stress, and environmental changes are most pronounced in the first 3 to 5 days of hospitalization, particularly within the first three days. As hospital stays extend, patients typically adjust to these changes (26). Consequently, the lowest sleep quality scores were recorded on the third day. To mitigate these effects, nurses should intensify patient education on disease management and psychological support early in hospitalization to alleviate anxiety and minimize the adverse impacts of stress on sleep. Additionally, reducing nighttime medical activities, controlling noise from medical devices, and establishing a quiet ward environment are crucial for enhancing patient comfort.

### 4.2 The effect of LS on the trajectory of sleep disturbance in geriatric oncology inpatients

This study revealed that patients with LS exhibited lower overall sleep quality and were more likely to experience persistent sleep disturbances. Analysis of sleep disturbance trajectories in inpatients with varying LS statuses showed that in the non-LS group, 14.1% (40/284) had no sleep disturbances, 15.8% (45/284) showed improvement, and 7.7% (22/284) experienced persistent disturbances. In contrast, in the LS group, only 11.3% (32/284) had no sleep disturbances, 17.6% (50/284) showed improvement, and a significant 33.5% (95/284) suffered from persistent disturbances. This suggests that LS patients are more likely to suffer from persistent sleep issues. Vanderlinden noted that lower limb muscle strength and balance ability are closely linked to sleep quality, stronger musculoskeletal function generally correlates with better sleep quality. However, his study focused on individuals over 55 years old, which differs slightly from this study (27). Two primary reasons account for these findings: firstly, the difference in research subjects. The current study focuses on geriatric patients with cancer, where the tumor exacerbates physiological and pathological changes, thereby increasing the incidence of LS compared to the general elderly population. Different diseases affect LS uniquely due to their distinct pathological mechanisms, the incidence of movement disorders in cancer patients is higher than in other diseases.

Therefore, during patients’ hospitalization, nurses should not only focus on the effects of disease conditions, psychological states, and the unique hospital environment on sleep quality but also closely monitor patients’ LS. When necessary, performing LS assessments and implementing appropriate interventions to enhance LS levels is crucial. Such measures may include enhancing health education, fostering an atmosphere conducive to daily activities or exercise in the ward, and focusing on musculoskeletal health and sleep guidance. These efforts aim to improve sleep quality during hospitalization and facilitate disease recovery.

This study indicates that patients with LS experience more significant sleep disturbances. Based on this, clinicians may consider non-pharmacological interventions to improve the severity of LS, thereby indirectly enhancing sleep quality during hospitalization in geriatric cancer patients. Furthermore, since LS is an early indicator of MF, early intervention upon diagnosing LS may help patients establish awareness of MF management, potentially preventing the progression to frailty or sarcopenia.

## 5 Conclusion

In summary, this study demonstrated that sleep disturbances are prevalent among hospitalized geriatric oncology inpatients, with disturbances most pronounced on the third day after admission. A further comparison of sleep disturbance trajectories revealed that patients with LS exhibited lower overall sleep quality and were more likely to experience persistent sleep disturbances.

## 6 Limitations

This was a single-center study with a relatively small sample size and limited duration of sleep monitoring. Moreover, the study excluded acutely ill geriatric oncology inpatients and did not categorize or specifically analyze the types of tumors affecting the patients, which may limit the comprehensiveness of the results. Also, it’s critical to identify the factors contributing to poor sleep in this population, including antidepressant therapy, comorbid chronic conditions, specific medication use, etc. Efforts to refine these results will be made in future studies.

## Data Availability

The raw data supporting the conclusions of this article will be made available by the authors, without undue reservation.
